# Analysis of lead toxicity in human cells

**DOI:** 10.1186/1471-2164-13-344

**Published:** 2012-07-27

**Authors:** Bruce S Gillis, Zarema Arbieva, Igor M Gavin

**Affiliations:** 1 Department of Medicine, University of Illinois at Chicago, Chicago, IL, USA; 2 Research Resources Center, University of Illinois at Chicago, Chicago, IL, USA

**Keywords:** Lead, Heavy metals, Cytokines, Gene expression, Peripheral blood mononuclear cells, Zinc protoporphyrin

## Abstract

**Background:**

Lead is a metal with many recognized adverse health side effects, and yet the molecular processes underlying lead toxicity are still poorly understood. Quantifying the injurious effects of lead is also difficult because of the diagnostic limitations that exist when analyzing human blood and urine specimens for lead toxicity.

**Results:**

We analyzed the deleterious impact of lead on human cells by measuring its effects on cytokine production and gene expression in peripheral blood mononuclear cells. Lead activates the secretion of the chemokine IL-8 and impacts mitogen-dependent activation by increasing the secretion of the proinflammatory cytokines IL-6 and TNF-α and of the chemokines IL-8 and MIP1-α in the presence of phytohemagglutinin. The recorded changes in gene expression affected major cellular functions, including metallothionein expression, and the expression of cellular metabolic enzymes and protein kinase activity. The expression of 31 genes remained elevated after the removal of lead from the testing medium thereby allowing for the measurement of adverse health effects of lead poisoning. These included thirteen metallothionein transcripts, three endothelial receptor B transcripts and a number of transcripts which encode cellular metabolic enzymes. Cellular responses to lead correlated with blood lead levels and were significantly altered in individuals with higher lead content resultantly affecting the nervous system, the negative regulation of transcription and the induction of apoptosis. In addition, we identified changes in gene expression in individuals with elevated zinc protoporphyrin blood levels and found that genes regulating the transmission of nerve impulses were affected in these individuals. The affected pathways were G-protein mediated signaling, gap junction signaling, synaptic long-term potentiation, neuropathic pain signaling as well as CREB signaling in neurons. Cellular responses to lead were altered in subjects with high zinc protoporphyrin blood levels.

**Conclusions:**

The results of our study defined specific changes in gene and protein expression in response to lead challenges and determined the injurious effects of exposures to lead on a cellular level. This information can be used for documenting the health effects of exposures to lead which will facilitate identifying and monitoring efficacious treatments for lead-related maladies.

## Background

Lead (Pb) is a widely distributed industrial metal and it also is naturally present in the environment. It is an environmentally persistent element and a major global environmental hazard. The Centers for Disease Control (CDC) currently consider lead poisoning the leading environmental health threat to children in the US. Lead-based paint is a primary source of lead exposure and the major source of lead toxicity in children. Lead exposure also remains one of the leading causes of workplace illness [1].

Lead contamination mainly occurs through absorption *via* the respiratory and gastrointestinal systems. Approximately 30-40% of inhaled lead enters the bloodstream [2]. Once absorbed, 99 percent of lead is retained in the blood for approximately 30-35 days and over the following 4-6 weeks it is dispersed and accumulated in other tissues – liver, renal cortex, aorta, brain, lungs, spleen, teeth and bones [3]. The half-life of lead in brain tissue is about two years and in bones it persists for 20-30 years [4]. Liver tissue is the largest repository of lead (33%) followed by the kidney cortex and medulla [5]. Depending on the amount of exposure, lead can adversely affect the nervous system, kidneys, the immune system, reproductive and developmental systems and the cardiovascular system. Its toxic effects vary from subtle changes in neurocognitive function in low-level exposures to a potentially fatal encephalopathy in acute lead poisoning [1]. Infants and young children are especially sensitive to low levels of lead which may contribute to behavioral problems, learning deficits and lowered IQ.

The molecular mechanisms of lead toxicity are still not clearly defined. The effects of lead on calcium fluxes and calcium-regulated events have been suggested as major mechanisms of lead neurotoxicity [6-8]. Lead also stimulates calmodulin and cAMP phosphodiesterase and enhances calmodulin-mediated protein phosphorylation in synaptic vesicles ([4] and references therein), thereby interfering with calcium/calmodulin-mediated neurotransmitter release. Another potential mechanism of lead toxicity is the ability of lead to induce oxidative stress. The deleterious effects of lead exposures can involve both the generation of reactive oxygen or nitrogen species (ROS) and a direct depletion of the antioxidant reserves (reviewed in [9]). Lead decreases glutathione levels by directly binding to thiol groups and inhibiting glutathione reductase [10]. Lead also inhibits δ-aminolevulinic acid dehydrogenase (ALAD) resulting in increased levels of δ-aminolevulinic acid (ALA) which is known to stimulate ROS production [11]. Lead also can stimulate membrane lipid peroxidation by binding to phosphatidylcholine in the cellular membrane and inducing changes in membrane biophysical properties [12,13]. ROS production and the generation of other potentially genotoxic compounds are possible mechanisms of the carcinogenicity of lead [9].

The diagnosis of lead poisoning has traditionally relied on measuring blood lead and zinc protoporphyrin levels. It is commonly accepted that the blood level concentration is the single best indicator of recent lead exposure [14]. The U.S. Centers for Disease Control established a 10μg/dL lead concentration in blood as the concern limit for exposures in children [15]. The blood lead level rises within hours of exposure and remains elevated for several weeks thereafter [16]. Due to lead’s short half-life time in the blood, blood lead tests cannot be used to diagnose or rule out evidence of exposure that occurred more than six weeks before testing. Zinc protoporphyrin or free erythrocyte protoporphyrin accumulates in erythrocytes as a result of the inhibition of heme synthesis [17]. Protoporphyrin levels begin to rise when blood lead levels exceed 1.5 to 2 μM and remain elevated for several months after exposure [18]. The protoporphyrin test is not as sensitive as the direct measurement of lead levels as other inhibitors of heme biosynthesis or iron deficiency anemia also increase protoporphyrin levels [19]. The genetic deficiency of ferrochelatase, a heme biosynthetic enzyme may lead to inaccurate test results as well [20].

Measurements of lead in tissues are useful in estimating exposure doses but they do not document injurious effects from the amount of lead that was absorbed by the body. Consequently, a methodology to track and quantify the injurious effects of exposures to lead is not only desirable, it is critical in order to identify appropriate preventive and therapeutic methodologies. And because the injurious effects of lead are often not recognized until the disease has advanced, identifying subclinical effects of exposures to lead is vital for early intervention. In an attempt to identify the biological effects of exposures to lead as well as develop and test the methodology for evaluating lead's injurious consequences at a cellular level, we performed global gene expression profiling in human cells challenged to lead. In our recent studies, we have previously utilized this methodology to comprehensively assess unique responses to environmental toxins in cultured peripheral blood mononuclear cells (PBMC) [21,22]. In this study we identified the cellular processes and pathways which were affected by exposures to lead, as well as the nature of cellular defences against lead insults.

## Results

### Subjects characteristics

We recruited a group of 44 subjects who did not have any known occupational exposures to lead and none had ever been diagnosed with lead-related disorders. The serum lead levels in the group were in a range from 0.1 to 5.8 μg/dL, with an average of 2.13 μg/dL. ZPP levels in the blood of the subjects ranged from 15-101 μg/dL with an average of 42.7 μg/dL. Since normal levels for blood zinc protoporphyrin are defined as being in the range of 16-36 μg/dL [23], a significant portion of the test subjects had elevated ZPP test results.

### Cytokine expression

**Figure 1:**
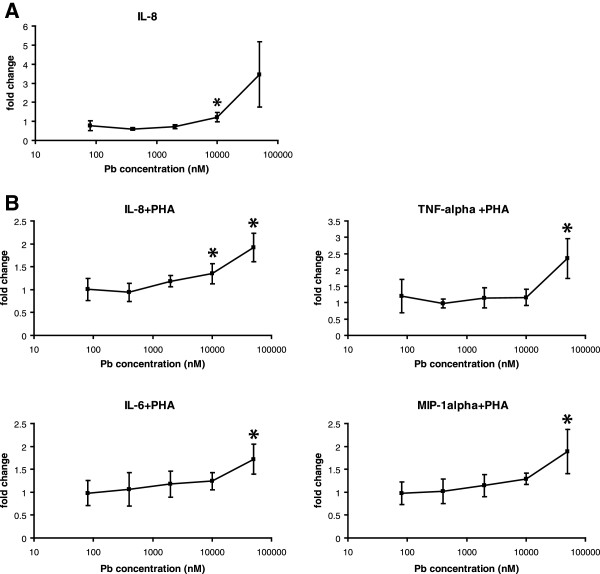
**Cytokine production by PBMC challenged to lead acetate.** Concentrations of cytokines in PBMC cultures challenged to either lead acetate alone at indicated concentrations (**A**) or to lead acetate in combination with 1μg/ml PHA (**B**) were normalized to those in control cultures. Statistically significant changes are marked with asterisks.

To determine how lead affects PBMC functions, we analyzed the impact of lead acetate on the secretion of 15 common cytokines by PBMC. The majority of the cytokines were below the lower detection limit and only chemokines IL-8, MIP-1β, MCP-1, Eotaxin, MIP-1α and RANTES were detected in the PBMC culture supernatants (not shown). Challenging cells with lead acetate at concentrations 10μM and above resulted in a dose-dependent increase of IL-8 production (Figure 1A). We also examined the effects of lead acetate on the mitogen-activated expression of cytokines. PHA stimulated production of all 15 cytokines in the control cultures which resulted in a significant increase in cytokine levels [21,22]. Lead acetate further increased the PHA-stimulated production of proinflammatory cytokines IL-6, TNF-α and chemokines IL-8, and MIP-1α (Figuref 1B). The highest increase of cytokine levels was at 50 μM lead acetate and some effect was also detected at 10 μM. These results demonstrated that lead increases mitogenic activation of PBMC, which is in agreement with the lead-dependent stimulation of lymphocyte and leukocyte proliferation and function observed in earlier studies [24-27].

### Gene expression profiling in PBMC challenged to lead

The cytokine expression study identified the specific impact of lead on mononuclear cell functions. To better understand how exposures to lead affect cellular processes on a molecular level and in order to identify intracellular responses to lead, we performed a genome-wide expression profiling in the PBMC of seven healthy individuals challenged to 10 μM lead acetate, the lowest concentration which affected cytokine production. Culturing cells in the presence of lead acetate for one day significantly altered the expression of 271 transcripts, of which expression of 221 transcripts changed 1.5-fold or more, including 157 downregulated transcripts and 64 upregulated transcripts. We observed elevated expressions of a number of cellular metabolic enzymes and metal-binding proteins which are important components in cellular defense responses against heavy metal insults. For example, of sixteen transcripts which were elevated 3-fold or more in lead-treated cells, twelve corresponded to seven metallothionein (MT) genes and the MT pseudogene. MT is a family of cysteine-rich, low molecular weight proteins which bind heavy metals through the thiol group of their cysteine residues [28]. MT proteins are implicated in protecting against metal-induced toxicity as well as oxidative stress and their expression is induced by a number of stimuli, including exposures to metals, oxidative stress, glucocorticoids, hydric stress and others [29,30].

To determine what cellular functions were most affected by the exposure to lead, we applied the DAVID functional bioinformatics resources tool to identify annotation clusters enriched with differentially expressed genes. Two annotation clusters containing methallothionein genes were identified, methallothioneins and cadmium/copper ion binding (Table 1). Metallothioneins and protein kinases were also among the functional groups of genes that were affected by exposure to lead (Table 2).

**Table 1:** Annotation clusters of genes affected by lead

**Annotation Cluster 1**	**Enrichment Score: 9.7**			
**Category**	**Term**	**Count**	**P-value**	**FDR**
UP_SEQ_FEATURE	metal ion-binding site: Divalent metal cation; cluster B	7	2.02E-10	3.01E-07
UP_SEQ_FEATURE	metal ion-binding site: Divalent metal cation; cluster A	7	2.02E-10	3.01E-07
PIR_SUPERFAMILY	PIRSF002564:metallothionein	7	2.15E-10	2.26E-07
INTERPRO	IPR018064: Metallothionein, vertebrate, metal binding site	7	2.23E-10	3.05E-07
SP_PIR_KEYWORDS	metal-thiolate cluster	7	3.86E-10	4.98E-07
INTERPRO	IPR000006: Metallothionein, vertebrate	7	4.43E-10	6.06E-07
INTERPRO	IPR003019: Metallothionein superfamily, eukaryotic	7	4.43E-10	6.06E-07
**Annotation Cluster 2**	**Enrichment Score: 5.8**			
**Category**	**Term**	**Count**	**P-value**	**FDR**
SP_PIR_KEYWORDS	cadmium	6	2.79E-09	3.59E-06
GOTERM_MF_FAT	GO:0046870 ~ cadmium ion binding	6	2.94E-08	4.01E-05
SP_PIR_KEYWORDS	copper	6	1.47E-04	0.19
GOTERM_MF_FAT	GO:0005507 ~ copper ion binding	6	7.95E-04	1.08

**Table 2:** Functional families of genes affected by lead

**Group1. IPR000006: Metallothionein, vertebrate**	**Enrichment Score: 6.0**
217546_at	metallothionein 1M
213629_x_at, 210524_x_at, 217165_x_at	metallothionein 1F
206461_x_at	metallothionein 1H
212185_x_at	metallothionein 2A
204745_x_at	metallothionein 1G
208581_x_at, 204326_x_at	metallothionein 1X
216336_x_at, 212859_x_at	metallothionein 1L (gene/pseudogene); metallothionein 1E; metallothionein 1 pseudogene 3; metallothionein 1J (pseudogene)
**Group 2. GO:0004672 protein kinase activity**	**Enrichment Score: 1.2**
211993_at	WNK lysine deficient protein kinase 1; hypothetical LOC100132369
244846_at	mitogen-activated protein kinase kinase kinase kinase 4
214464_at	CDC42 binding protein kinase alpha (DMPK-like)
241403_at	CDC-like kinase 4
213328_at	NIMA (never in mitosis gene a)-related kinase 1

### Residual effect of exposures to lead

Exposures to lead of human PBMC adversely affected their functions as evidenced by the changes in gene expression. However, these changes may reverse when lead is no longer available in the medium. To investigate the residual, adverse effects of lead poisoning on the cells, we removed lead acetate from the cell cultures and determined the persistent changes in the gene expression. After an initial incubation with lead acetate for one day, the cells were washed three times and cultured in fresh RPMI medium without lead acetate for one additional day, for a total of two days. Gene expression in these cells from the entire group of 44 individuals was compared with control cells from the same group which were cultured under the identical conditions in the absence of lead acetate. We identified 604 transcripts whose expressions were different in these two groups of cells. 146 transcripts corresponded to 104 genes whose expression increased 1.5-fold or more in lead-challenged cells, and 58 transcripts corresponded to 39 genes whose expressions decreased 1.5-fold or more in exposed cells. The observed changes were consistent with cells recovering from the lead insult and the restoration of their functions. However, it is likely that the changes in the expression of many genes resulted from removing lead from the medium. In order to identify genes whose expressions changed in response to the lead challenge and remained altered after removing lead from the cultures, we compared the lead-dependent changes in one-day cultures and in two-day cultures. We identified 42 transcripts, corresponding to 31 genes whose expressions changed similarly in both groups. Expressions of 38 transcripts corresponding to 22 genes were at least 2 times higher than in the control cells. The list of these genes is shown in Table 3. Eighteen transcripts remained elevated three-fold or more in lead-exposed cells after removing lead from the culture. We observed elevated levels of all 12 MT transcripts, the pirin transcript (an iron-binding nuclear protein), three endothelial receptor type B transcripts, aldehyde dehydrogenase 1, the KIT ligand and the tryptophan 2,3-dioxygenase transcripts. Other transcripts included four transcripts encoding dihydrodiol dehydrogenase, six transcripts encoding aspartate beta-hydroxylase, four transcripts encoding solute carrier proteins, two transcripts encoding prostaglandin reductase, transcripts encoding arrestin, and malic enzyme. These genes represented a panel of putative cellular biomarkers which were induced by lead and whose expressions remained activated after lead was removed from the medium. Since the changes in expression of these genes were not transient, they could be utilized for evaluating exposures to lead.

**Table 3:** Persistent changes in gene expression induced by lead

**Gene Symbol**	**Gene Title**	**Probe Set ID**	**Entrez Gene ID**	**Fold Increase**	**P-value**
MT1M	metallothionein 1M	217546_at	4499	18	1.03E-22
MT1G	metallothionein 1G	204745_x_at	4495	17.7	1.64E-27
MT1H	metallothionein 1H	206461_x_at	4496	14.2	6.96E-28
MT1X	metallothionein 1X	208581_x_at	4501	13.9	1.90E-26
		204326_x_at	4501	13	1.66E-25
MT1E	metallothionein 1E	212859_x_at	4493	10.9	4.23E-26
MT2A	metallothionein 2A	212185_x_at	4502	10.6	2.93E-27
MT1P2	metallothionein 1 pseudogene 2	211456_x_at	645745	9.55	2.96E-27
MT1F	metallothionein 1F	217165_x_at	4494	8.86	1.29E-24
		213629_x_at	4494	6.6	2.47E-21
		210524_x_at	4494	4.62	2.72E-19
MT1E///MT1H///MT1M///MT1P2	metallothionein 1E///metallothionein 1H///metallothionein 1M///metallothionein 1 pseudogene 2	216336_x_at	4493///4496///4499///645745	7.44	4.60E-23
EDNRB	endothelin receptor type B	204273_at	1910	11	1.11E-17
		206701_x_at	1910	6.6	8.42E-13
		204271_s_at	1910	5.99	3.32E-13
ALDH1A1	aldehyde dehydrogenase 1 family, member A1	212224_at	216	4.37	6.93E-06
KITLG	KIT ligand	226534_at	4254	3.19	2.02E-09
TDO2	tryptophan 2,3-dioxygenase	205943_at	6999	3.14	0.004728
TMEM158	transmembrane protein 158	213338_at	25907	2.96	6.16E-15
FAM70A	family with sequence similarity 70, member A	219895_at	55026	2.87	0.000112
PTGR1	prostaglandin reductase 1	231897_at	22949	2.86	3.65E-08
		228824_s_at	22949	2.74	4.81E-07
ARRDC4	arrestin domain containing 4	225283_at	91947	2.8	7.31E-11
AKR1C1	aldo-keto reductase family 1, member C1 (dihydrodiol dehydrogenase 1; 20-alpha)	204151_x_at	1645	2.68	0.000934
		216594_x_at	1645	2.28	3.79E-05
AKR1C2	aldo-keto reductase family 1, member C2 (dihydrodiol dehydrogenase 2; bile acid)	211653_x_at	1646	2.47	4.10E-05
		209699_x_at	1646	2.28	5.27E-05
ME1	malic enzyme 1, NADP(+)-dependent, cytosolic	204058_at	4199	2.53	9.73E-07
		204059_s_at	4199	2.3	9.20E-08
SLC7A11	solute carrier family 7, (cationic amino acid transporter, y + system) member 11	207528_s_at	23657	2.58	3.45E-07
		209921_at	23657	2.42	7.46E-06
		217678_at	23657	2.37	4.72E-05
ASPH	aspartate beta-hydroxylase	210896_s_at	444	2.41	1.80E-11
		209135_at	444	2.5	3.04E-10
		224996_at	444	2.08	9.56E-08
		225008_at	444	1.87	4.93E-08
		205808_at	444	1.58	7.60E-05
		242037_at	444	1.57	1.32E-06
ENSG00000204134	---	238727_at	---	2.23	0.000495
SLC12A8	solute carrier family 12 (potassium/chloride transporters), member 8	219874_at	84561	2.17	1.85E-08
PIR	pirin (iron-binding nuclear protein)	207469_s_at	8544	2.01	0.000561

### Cellular responses to lead in subjects with elevated blood lead levels

A number of studies have demonstrated that lead exposures resulting in blood lead levels below 10 μg/dL may cause cognitive dysfunction, neurobehavioral disorders, neurological damage, hypertension and renal impairment [31-33]. To determine if cellular responses to lead are altered in individuals with elevated blood lead levels which are still below the safety concern threshold level, we divided the subjects into three groups based on their lead test results and compared gene expressions between these groups. The control “low lead” group consisted of eight individuals whose blood lead levels were equal to or below 1μg/dL, with a 0.7 μg/dL mean value. The “moderate lead” group had nine individuals with blood lead levels ranging from 2 μg/dL to 3 μg/dL, with a 2.6 μg/dL mean value and the “high lead” group contained seven subjects with lead test results above 4 μg/dL. The lead levels in this group ranged from 4.4 μg/dL to 5.8 μg/dL and the mean value was 5.2 μg/dL. Initially, we compared gene expressions in the control cultures to determine the basal differences in the gene expression pattern between the groups. Gene expressions in the “moderate lead” and “high lead” group were similar to the “low lead” group. There was no statistically significant difference in the gene expression patterns between the “moderate lead” and the “low lead” groups and only one transcript was identified which was upregulated in the “high lead” group. However, when the cells were challenged to lead acetate, marked changes in gene expressions were observed between these groups depending on the blood lead levels. Expressions of 17 transcripts in the “moderate lead” group and expressions of 1440 transcripts in the “high lead group” were altered compared to the “low lead” group. Eight transcripts in the “moderate lead” set of 17 differentials were also present among 1440 transcripts identified in the “high lead” group. Annotation clustering revealed that the most affected cellular processes were negative regulation of transcription and the induction of apoptosis (Table 4). Functional analysis of the gene expression data showed that nervous system development and function were the most affected categories in the "high lead" group (Table 5). These results show that the responses of the cells were significantly altered in individuals with higher blood lead levels affecting primarily the nervous system and supported the view that the blood lead levels lower than the CDC-recommended concern level have deleterious effects on cellular functions.

**Table 4:** Annotation clusters affected by lead in individuals with high blood lead levels

**Annotation Cluster 1**	**Enrichment Score: 4.6**			
**Category**	**Term**	**Count**	**P-value**	**FDR**
GOTERM_BP_FAT	GO:0010941 ~ regulation of cell death	84	2.05E-05	0.04
GOTERM_BP_FAT	GO:0042981 ~ regulation of apoptosis	83	2.21E-05	0.04
GOTERM_BP_FAT	GO:0043067 ~ regulation of programmed cell death	83	3.15E-05	0.06
**Annotation Cluster 2**	**Enrichment Score: 4.4**			
**Category**	**Term**	**Count**	**P-value**	**FDR**
GOTERM_BP_FAT	GO:0010629 ~ negative regulation of gene expression	59	1.24E-05	0.02
GOTERM_BP_FAT	GO:0016481 ~ negative regulation of transcription	55	1.33E-05	0.02
GOTERM_BP_FAT	GO:0010558 ~ negative regulation of macromolecule biosynthetic process	62	1.97E-05	0.04
GOTERM_BP_FAT	GO:0031327 ~ negative regulation of cellular biosynthetic process	63	2.22E-05	0.04
GOTERM_BP_FAT	GO:0009890 ~ negative regulation of biosynthetic process	64	2.23E-05	0.04
GOTERM_BP_FAT	GO:0045934 ~ negative regulation of nucleobase, nucleoside, nucleotide and nucleic acid metabolic process	57	7.26E-05	0.13
GOTERM_BP_FAT	GO:0051172 ~ negative regulation of nitrogen compound metabolic process	57	1.05E-04	0.19
GOTERM_BP_FAT	GO:0010605 ~ negative regulation of macromolecule metabolic process	71	6.31E-04	1.14
**Annotation Cluster 3**	**Enrichment Score: 4.0**			
**Category**	**Term**	**Count**	**P-value**	**FDR**
GOTERM_BP_FAT	GO:0043068 ~ positive regulation of programmed cell death	50	9.01E-05	0.16
GOTERM_BP_FAT	GO:0010942 ~ positive regulation of cell death	50	1.00E-04	0.18
GOTERM_BP_FAT	GO:0043065 ~ positive regulation of apoptosis	49	1.47E-04	0.27
**Annotation Cluster 4**	**Enrichment Score: 3.8**			
**Category**	**Term**	**Count**	**P-value**	**FDR**
GOTERM_BP_FAT	GO:0016481 ~ negative regulation of transcription	55	1.33E-05	0.02
GOTERM_BP_FAT	GO:0045892 ~ negative regulation of transcription, DNA-dependent	41	4.50E-04	0.81
GOTERM_BP_FAT	GO:0051253 ~ negative regulation of RNA metabolic process	41	6.25E-04	1.13

**Table 5:** Physiological processes affected by lead in individuals with high blood lead levels

**Category**	**Count**	**P-Value**
Nervous system development and function	8	8.32E-04 - 4.62E-02
Proliferation of Schwann cells	3	8.32E-04
Growth of axons	3	2.50E-02
Axonal transport	2	4.62E-02
Tissue development	18	3.62E-03 - 4.62E-02
Tumor Morphology	9	6.34E-03 - 4.62E-02
Hematological system development and function	22	9.70E-03 - 4.81E-02
Hematopoiesis	13	9.70E-03 - 3.61E-02

### Gene expression profiling in subjects with elevated ZPP test results

**Figure 2:**
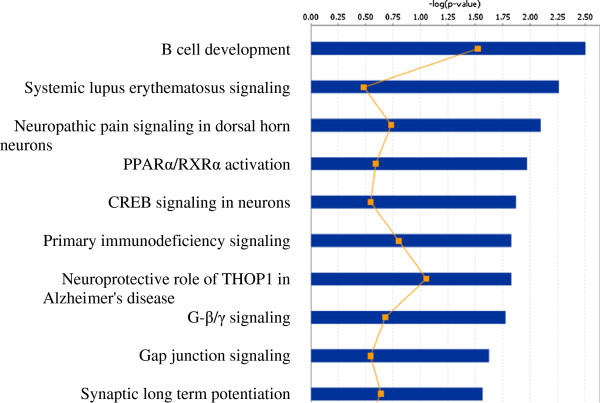
**Physiological processes affected by lead challenge in subjects with high blood ZPP content.** The categories were sorted by their scores (blue bars). Yellow graph corresponds to the ratio of the number of affected genes to the total number of genes in this category.

One of the inclusion parameters for selecting a cohort of 44 subjects was the normal blood lead test result. Unlike blood lead levels which decrease to normal in several weeks after exposure, blood ZPP levels remain elevated for several months [18]. Zinc protoporphyrin levels in the blood samples of 28 subjects were above the normal range. It is possible that some individuals may have been unknowingly exposed to lead in the past and their blood lead levels returned to normal at the time the test was taken. To explore this possibility, we determined whether cellular responses to lead were altered in the subjects with higher ZPP levels. We placed the subjects in two groups according to their ZPP test result. The “high ZPP” group contained fifteen subjects whose ZPP levels were above normal. In this group, the ZPP test results ranged from 43 μg/dL to 101 μg/dL and the mean value was 55 μg/dL. Fifteen subjects in the “normal ZPP” group had ZPP levels in the normal range, from 20 μg/dL to 35 μg/dL, with the mean value of 28 μg/dL. The mean blood lead levels in both groups were 3 μg/dL and 1.1 μg/dL, respectively, and the difference was statistically significant. Initially, we determined the difference in gene expressions in control cells. We identified 567 transcripts whose abundance was different in these two groups. For most transcripts, the ratios of the signals were below 1.5 and the levels of only 140 transcripts changed 1.5-fold or more. Among the genes that were downregulated in the “high ZPP” group were angiopoietin genes as well as genes encoding cyclin 1 and fibroblast growth factor receptor 2. The expressions of these genes were also inhibited in human cell lines challenged to ZPP [34]. DAVID annotation clustering analysis revealed that the most affected cellular process in the “high ZPP” group was the positive regulation of synaptic transmission (Table 6). Pathway analysis also identified a number of groups related to the nervous system. Among the groups with top ten scores were neuropathic pain signaling, CREB signaling in neurons, neuroprotection in Alzheimer's disease, G_βγ_ signaling, gap junction signaling, and synaptic long term potentiation (Figure 2). Other groups were related to the immune system.

**Table 6:** Annotation clusters of genes affected in individuals with higher ZPP levels

**Annotation Cluster 1**	**Enrichment Score: 2.8**				
**Category**	**Term**	**Count**	**P-Value**	**Fold Enrichment**	**FDR**
GOTERM_BP_FAT	GO:0050806 ~ positive regulation of synaptic transmission	6	0.0013	7.3	2.1
GOTERM_BP_FAT	GO:0051971 ~ positive regulation of transmission of nerve impulse	6	0.0018	6.7	3.1
GOTERM_BP_FAT	GO:0031646 ~ positive regulation of neurological system process	6	0.0023	6.3	3.9

To determine if the cells from the subjects with elevated ZPP levels responded differently to the lead challenges, we compared gene expressions in the “high ZPP” group and the “normal ZPP” group in the cell samples challenged to lead. A total of 1230 transcripts were identified whose signals were different in the two study groups by a statistically significant value. Of those, only 275 transcripts had a ratio of mean signal values higher than 1.5. 146 transcripts in this group were also identified in control cultures at the 10% confidence level. Expressions of the majority of genes therefore varied between these two groups regardless of a lead challenge. A remaining 129 transcripts were found only in the cells challenged to lead and signified the difference in the responses to lead between the “high ZPP” group and the “normal ZPP” group (Additional file 1: Table S1). These results demonstrated that cellular responses to lead were altered in subjects with elevated blood ZPP levels.

## Discussion

For many chronic diseases, the specific role of environmental exposures in causing diseased phenotypes is not well understood. It is vital to determine when injurious exposures have occurred long before there is the appearance of overt clinical signs and symptoms in humans. Because these injurious effects most certainly develop in a discrete cellular manner, we applied gene expression and protein profiling to find and categorize these cellular effects. Cytokine profiling, in particular, is very important for understanding how environmental exposures affect the expression of these soluble mediators which are produced in tissues undergoing defence, growth, differentiation and repair processes [35]. Even low-level exposures to lead impair cell-mediated immunity by upsetting the balance between Th1- and Th2- like T lymphocytes which alters cytokine expression [36-38]. The changes in proinflammatory cytokines also play a role in the neurotoxicity of lead [39-41]. We demonstrated that lead exposures resulted in a dose-dependent increase of IL-8 production by PBMC as well as enhanced the production of the proinflammatory cytokines IL-6, TNF-α and the chemokines IL-8 and MIP-1α in response to mitogens. The increase in the TNF-α level was in agreement with previous studies [42] and was consistent with epidemiological data on lead-exposed workers [43]. Our observation that lead increases mitogenic activation of PBMC was in agreement with the activation of lymphocyte and leukocyte proliferation and function by lead which has been observed in earlier studies [24-27]. One mechanism by which lead may activate PBMC is by inducing PKC-dependent cell signaling cascades [4]. Our findings demonstrated that exposure to lead strongly affects cell signaling pathways, particularly ATP binding and kinase activity.

In an attempt to find changes in gene expression following exposures to lead, we identified genes whose expressions were induced by lead and remained activated even after the lead was removed from the media. The most pronounced effect was the activation of the expression of metallothionein (MT) genes. MT gene expression has been identified and utilized as a biomarker of heavy metal exposures in a variety of biological systems [44-51] including in human cell lines [52] as well as in exposures to cadmium in humans [53]. Our findings imply that expressions of metallothionein genes in human tissues also may be applied to assessing exposures to lead. Another gene whose expression was activated by lead was the endothelin receptor B (EDNRB). Although the mechanism of EDNRB regulation by lead is unknown, an increase of its expression may contribute to the hypertension observed in low-level lead exposures [54].

A number of metabolic enzymes were also upregulated by lead exposures, including the genes encoding aldehyde dehydrogenase, tryptophan 2,3-dioxygenase, malic enzyme, aspartate beta-hydroxylase, prostaglandin reductase 1 and two members of the aldo-keto reductase family 1, C1 and C2. All of these enzymes with the exception of tryptophan 2,3-dioxygenase and aspartate beta-hydroxylase are NAD(P) + dependent. Tryptophan 2,3-dioxygenase catalyzes the degradation of tryptophan into N-formyl-kynurenine. Tryptophan depletion and the accumulation of its metabolites induce cell cycle arrest and apoptosis of T lymphocytes, thereby suppressing T-cell activation [55,56]. They also promote the differentiation of naive CD4+ T cells into regulatory T cells. An increase in the expression of this enzyme as a result of exposure to lead may explain the depletion of CD4+ T cells observed in exposed individuals [57-60]. An increased production of tryptophan metabolites was also implicated in neurodegenerative disorders [61]. For example, an increase of tryptophan 2,3-dioxygenase expression was found in astrocytes of schizophrenic patients [62]. Lead-dependent activation of the gene encoding this enzyme may contribute to the neurocognitive symptoms which are observed in lead-exposed individuals. Another possible mechanism by which exposures to lead can impair neurocognitive function is the dysregulation of neurosteroids. The Aldo-keto reductase family 1 enzymes, AKR1C1 and AKRC2 are implicated in steroid metabolism. AKRC1 inactivates progesterone by forming 20α-hydroxyprogesterone [63,64]. Progesterone and its neuroactive metabolites have modulatory effects on brain function and influence social, cognitive, emotional and physical processes [65,66]. Increased expression of AKR1C as a result of exposures to lead may change the levels of progesterone and its neuroactive metabolites, such as 3α,5α-THP, thereby contributing to neurocognitive disorders. Upregulation of the xenobiotic metabolizing enzymes AKR1C1 and AKRC2 caused by lead was also observed in primary normal human bronchial epithelial cells [67].

Among the enzymes that were upregulated in the cells challenged to lead were prostaglandin reductase 1, which inactivates leukotriene B4; 15-ketoprostaglandins which are important mediators of inflammation [68,69]; aspartate beta-hydroxylase, which plays an important role in calcium homeostasis [70]; malic enzyme, a cytosolic, NADP-dependent enzyme which generates NADPH for fatty acid biosynthesis [71]; and a cytosolic isoform of aldehyde dehydrogenase 1, an enzyme in the major oxidative pathway of alcohol metabolism [72]. Other activated genes included transcriptional cofactor pirin, an iron-binding nuclear protein involved in apoptosis [73]; two members of the amino acid/polyamine transport system, SLC12A8A [74] and SLC7A11 [75]; KIT ligand, the ligand of the tyrosine-kinase receptor encoded by the KIT locus, implicated in pigmentation [76] and cancer [77]; α-arrestin 4 [78]; transmembrane protein TMEM158; FAM70A and putative protein ENSG00000204134. Our findings that expressions of these genes were not transient and remained elevated even after the lead was removed from the system, demonstrate that they could be utilized as biomarkers in tests for assessing exposures to lead.

The U.S. Centers for Disease Control set a threshold for a significant exposure to lead at blood lead levels of 10 μg/dL or above which have an increased risk for both subclinical and overt effects from this substance [15]. In particular, individuals with a blood lead level of 10 μg/dL or above are at high risk for peripheral neuropathies and/or chronic nephropathies, with the latter often triggering consequential hypertension. The analysis of the gene expressions demonstrated that cellular responses to lead were significantly altered in individuals with blood lead levels around 5 μg/dL, suggesting that doses lower than 10 μg/dL impair cellular functions. The most affected system was the nervous system and the most affected groups were the negative regulation of transcription and the positive regulation of apoptosis. The changes in gene expressions correlated well with the blood lead levels and were in agreement with previous studies in children [79].

Although the blood lead test provides accurate information on lead absorption in persons with brief acute exposures, it cannot be used for assessing past or chronic exposures. It is well known that exposures to lead are also associated with elevated blood ZPP levels which remain elevated for several months after the exposure. The analysis of blood chemistries in 44 subjects demonstrated that elevated blood ZPP levels correlated with higher blood ZPP content. Expressions of genes encoding proteins involved in the transmission of nerve impulses were significantly affected in the “high ZPP” group. Among the affected pathways were G-protein mediated signaling, gap junction signaling, synaptic long-term potentiation, neuropathic pain signaling as well as CREB signaling in neurons (Figure 2). Therefore, the nervous system was highly affected in these individuals confirming clinical observations [31-33].

Blood ZPP levels are increased in individuals who are deficient in mineral iron or metabolism [19,80], therefore ZPP measurements only provide indirect evidence of an exposure to lead. Nevertheless, the cellular responses to lead were changed in the subjects with the elevated blood ZPP levels which were signified by the altered gene expression profiles.

## Conclusions

The present study defined discrete and unique responses to lead in cultured PBMC. Our findings define the injurious effects from lead exposures at the cellular level. The methodology of detecting the health effects of environmental exposures based upon individual cellular response patterns offers a starting point for assessing injurious consequences of such exposures and to developing appropriate health treatment protocols. This approach makes it possible to obtain human toxicity data which can be used to identify the potentially injurious effects of exposures to any occupational and environmental compound for which human toxicity data are not yet available. Human medical toxicology is based upon the appreciation that after target cells of a toxin are impacted, there are two primary phases of reaction. One is composed of the acute side effects, which are then followed by the chronic and residual impact of the toxin. We have been able to document both the “acute” and “residual” effects of lead on human cells based upon changes in gene expression patterns. We believe that gene expression profiling can be utilized whenever there is a desire to confirm lead poisoning and it therefore merits being an adjunct to blood lead testing in lead-exposed individuals, particularly in those in which an adverse impact needs to be ascertained.

## Methods

### Human subjects

The study was approved by the Institutional Review Board Services, Aurora, Ontario, Canada. We recruited 44 healthy volunteers from the Los Angeles, CA area. Volunteers were of both genders ranging in age between 18 and 54 and equally distributed among Caucasians, Hispanics, African Americans and Asians. The subjects signed the consent forms, completed the health questionnaire forms and underwent physical examinations. Those who had no history of known personal or occupational exposures to lead and no background of lead-related symptoms or disorders underwent blood tests to determine their plasma lead and ZPP levels. The subjects whose plasma lead levels were in the normal range below 10 μg/dL were asked to provide 50 ml of blood for experimentation.

### Isolation and culture of PBMC

The blood was collected in (K3) EDTA collection tubes by venipuncture after obtaining appropriate informed consent. PBMC were isolated by Ficol gradient centrifugation as described earlier [21,22] and were suspended at 10^6^ cells/ml in RPMI 1640 medium supplemented with 1% penicillin-streptomycin, 1% L-glutamine and 10% fetal bovine serum (Invitrogen, Carlsbad, CA). The cells were cultured for 18 hours in three replicate plates in the presence of lead acetate at five concentrations: 0.08 μM, 0.4 μM, 2 μM, 10 μM and 50 μM either with or without 10μg/ml phytohemagglutinin (PHA-P, Sigma-Aldrich®, St. Louis, MO). Control cells were cultured in three replicate wells in the medium which did not contain lead. Lead concentrations were far below the toxicity levels observed in PBMC at concentrations above 500 μM [81].

### Cytokine assay

Cell culture supernatants were collected and concentrations of 15 common cytokines GM-CSF, IFN-γ, IL-1β, IL-2, IL-4, IL-5, IL-6, IL-8, IL-10, TNF-α, MIP-1β, MCP-1, Eotaxin, MIP-1α and RANTES were measured by using multiplex immunoassays based on Luminex xMAP™ bead array technology as described earlier [21,22]. PBMC isolated from three donors were used for the cytokine analyses. Mean concentration values of cytokines in cultures challenged to lead acetate were normalized to those in control cultures. The normalized values at 10 μM and 50 μM lead acetate were compared to those at 0.08 μM and 0.4 μM lead acetate by pooled t-tests. The variances in the groups were confirmed by the F-test. All F-test p-values were above 0.1, except for IL-8 at 50 μM lead acetate. T-test with the Satterthwaite approximation to the degrees of freedom was used for this challenge. All t-tests assumed two-tailed distribution and the confidence level was set at 5%.

### RNA isolation and gene array analysis

Total RNA was purified from PBMC samples by using Trizol reagent and RNeasy RNA purification kits (Qiagen Sciences, Valencia, CA) according to the manufacturer's instructions. Whole genome expression profiling was performed by using Human Genome U133 Plus 2.0 GeneChip® arrays (Affymetrix, Santa Clara, CA) interrogating over 47,000 transcripts from approximately 40,000 annotated human genes. All labeling reactions and hybridizations were carried out according to the Affymetrix GeneChip® eukaryotic target labeling protocol. Bound probes were detected by laser excitation of the fluorescent markers and the resultant emission spectra were obtained by using the Gene Array Scanner 3000 (Agilent Technologies, Santa Clara, CA). Data acquisition was performed using GCOS (Affymetrix GeneChip Operating Software Package). Data normalization, background correction and all subsequent statistical tests for significant differential expressions were performed by using a Partek statistical software package (Partek., Inc, St. Louis, MO). Data was normalized *via* a quintiles normalization and summarized using the Robust Multi-array Average (RMA) method [82]. The ANOVA test was used to calculate the significance of the differential expression between treated and untreated samples. Raw p-values were corrected for FDR (false discovery rate) using the Benjamini and Hochberg procedure [83] and a cut-off level equal or less than 0.05 was applied. Cluster analysis was performed by using the DAVID functional annotation tool [84]. Additional functional analysis was performed by using an Ingenuity Pathway Analysis software (Ingenuity Systems, Redwood City, CA). Gene array data were deposited in the Gene Expression Omnibus database, accession number GSE37567 [85].

## Abbreviations

PBMC, Peripheral blood mononuclear cells; ZPP, Zinc protoporphyrin; MT, Metallothionein; PHA, Phytohemagglutinin.

## Competing interests

The authors declare that they have no competing interests.

## Authors' contributions

BSG conceived of the study, designed it, recruited the patients and drafted the manuscript; IMG participated in the design of the study and its coordination, carried out the immunoassays, analyzed and interpreted the data and drafted the manuscript. ZA carried out the gene expression studies, and helped drafting the manuscript. All authors read and approved the final manuscript.

## Supplementary Material

Additional file 1 Changes in cellular responses to lead in individuals with high blood ZPP levels. Click here for file
